# Influence of Strengthening Material Behavior and Geometry Parameters on Mechanical Behavior of Biaxial Cruciform Specimen for Envelope Material

**DOI:** 10.3390/ma12172680

**Published:** 2019-08-22

**Authors:** Zhipeng Qu, Houdi Xiao, Mingyun Lv, Xihe Wang, Pengfei Wang, Lei Xu

**Affiliations:** 1 School of Aeronautic Science and Engineering, Beihang University, Beijing 100083, China; 2 Aviation Industry Corporation (AVIC) Special Vehicle Research Institute, Jingmen 448035, China

**Keywords:** cruciform, variation coefficient, stress concentration factor, geometry parameters

## Abstract

The stratospheric airship envelope material is operated in biaxial stress, so it is necessary to study the in-plane biaxial tensile strength. In this paper, a theoretical model is developed to evaluate the mechanical properties of in-plane biaxial specimens. Being applied to the finite element analysis, the theoretical model is employed to evaluate the influence of strengthening material behavior (E*) and geometry parameters on the mechanical behavior in the central. The follows results are drawn: (i) smaller the length of the central region (L_cen_), E* and larger the central region corner radius (r) contribute to smaller coefficient of variation (CV); (ii) smaller L_cen_ and larger E* contribute to smaller stress concentration factor (k), k in the limit state of r is larger than that in other conditions. (iii) The CV and k under stress ratio of 1:1 are smaller than those under other stress ratios. The study can provide a useful reference for the design of biaxial specimens.

## 1. Introduction

The stratospheric airship has attracted widespread attentions with potential applications in radio relay telecommunications service, earth observation science, and other fields [1,2,3,4]. Envelope material is a major part of airship structure. The mechanical properties and service life for envelope material determine the lifetime of airship [5,6]. The typical envelope of the non-rigid airship structure is laminated fabric composites [7]. The laminated material is used to contain lifting gas and provide structural strength for the system.

The uniaxial tensile test has been widely used to study the mechanical properties of envelope materials. Meng et.al [8] developed a theoretical model to predict fatigue life on envelope material under uniaxial cycle load. The theoretical model is in good agreement with the experimental results. Hu et al. [9] studied the mechanics of plain woven fabric URETEK 5893 under uniaxial monotonic and cyclic load in on-axis and off-axis tension. Meng et al. [10] studied the mechanical properties and the strength criteria of envelope material under uniaxial tension. Jin-Ho Roh et al. [11] investigated the thermoelastic behaviors of the stratospheric airship envelope by experimental and numerical simulation. Chen et al. [12] studied the elastic constants of envelope fabric Uretek3216A under mono-uniaxial, uniaxial cyclic, and biaxial cyclic loading. Chen Jianwen et al. [13] studied the mechanical behaviors of URETEK-3216LV material under mono-uniaxial, uniaxial cyclic, and biaxial cyclic loading. Liu et al. [14] developed a new physics model to calculate the tearing mechanical behavior of envelope material under uniaxial tension tests. Vannucchi de Camargo et al. [15] estimated an impact numerical simulation to exhibit a fracture mechanism. The finite element simulation describes a multiphase in detail. Cristiano Fragassa et al. [16] performed an analysis of the mechanical and impact properties of flax and basalt fibres by experimental investigation. 

In recent years, the mechanical properties of envelope materials have been gradually increased by biaxial tensile test. Chen et al. [17] studied the mechanical properties of PVC materials by biaxial tension. The results show that the tensile behavior of the coated membrane materials was dramatically affected by the stress ratio in the warp and fill directions. Chen et al. [12] studied the tensile properties of envelope fabric Uretek-3216A under biaxial cyclic loading; the results show that the elastic constants noticeably vary with the experimental protocols. Meng et al. [10] studied the strength of new envelope material under biaxial tension tests. Qu et al. [18] reported the constitutive model of the envelope material under different stress ratios and verified by biaxial experiments. Yasuhiro Hanabusa et al. [19] proposed a new method for evaluating stress measurement errors in biaxial tensile tests while using a cruciform specimen by numerical simulation. The new method was used to design uniformly thick biaxial tensile specimens of flat sheet metal and the design size range of the specimens was also given. Shi et al. [20] reported a new specimen design and test method for biaxial tensile strength. The biaxial tensile strength and uniaxial tensile strength were also compared and analyzed. The biaxial tensile strength under 1:1 load ratio equals uniaxial strength multiplied by an amplification factor of 1.1–1.3. Zhang et al. [21] studied the fracture failure analysis and failure criterion for Poly tetra fluoroethylene (PTFE)-coated woven fabric. On-axial and off-axial tensile tests of PTFE were carried out and biaxial tensile tests were also carried out. A new failure criterion was proposed and then verified by experiments. Alan Hannon et al. [22] published a review of biaxial tensile test devices and specimen design for biaxial testing. The advantages and disadvantages of biaxial tension test device and sample form were also evaluated and analyzed. R. Xiao [23] published a review of biaxial tensile testing technology and suggested new ideas for future development. The biaxial tension test equipment and advanced measurement methods were discussed. The stress-strain curves of sheet metals, the progress of finite element simulation analysis, and the research progress of biaxial specimen design are reviewed.

It is interesting to note in the above reviews that many scholars have contribution to biaxial tensile mechanical properties of flexible envelope materials from experimental and simulation. At present, two layers of the same material are used to strengthen the arm of the biaxial specimen, and one layer is used in the center part of the biaxial specimen to ensure that the failure occurs in the center section. However, the influence of the different modulus of strengthening material and geometry parameters of central test section on the mechanical properties of the biaxial specimen is rare studied. In this paper, the effects of modulus of different strengthening materials (based material: Uretek-3216LV) and different geometrical sizes in the central region on the mechanical properties of envelope materials are studied by using commercial software ABAQUS, based on the existing cruciform specimens.

This paper aims to develop a theoretical model for evaluating the effects of modulus and geometry parameters of the central region of reinforced materials on the mechanical properties of envelope materials. The structure of this paper is as follows: In Section 2, a theoretical model developed and envelope material is presented. In Section 3, biaxial specimen shape and analysis conditions are presented. In Section 4, commercial software ABAQUS was used to study the influence of strengthening material behavior and geometry parameters on mechanical behavior of biaxial cruciform specimen for envelope material are presented. In Section 5, a brief summary and conclusions are presented. The study can provide a reference for the biaxial tensile specimens of flexible envelope materials and serve the design of flexible structures.

## 2. Methodology

### 2.1. Theoretical Model

The shape and dimension of envelope material specimen have great influence on the experimental results. Reasonable specimen can ensure the validity of the tensile test. A new theoretical model is proposed in order to obtain a successful specimen. To satisfy a successful biaxial tensile test, the shape and sizes of biaxial tensile specimens has to meet the following requirements [24,25,26,27,28]: “(i) Maximization of the region of stress and strain uniformity in the biaxial loaded zone; (ii) Minimization of the shear strain in the biaxial load testing zone; (iii) Minimization of the stress and strain concentrations outside the testing zone; (iv) Specimen failure in the biaxial load testing zone; and, (v) Repeated results of the experiments”. It has been proven to be difficult to design cruciform specimens that simultaneously satisfy all of these requirements.

S. Demmerle et al. [26] have contributed to the criterion of biaxial tension. The theory contains nine evaluation parameters, and the evaluation of biaxial specimens is complicated. An evaluation theoretical model is developed in order to evaluate the quality of biaxial design sample conveniently and concisely. The theoretical model is as follows:

(i) Stress Variability Coefficient

The stress variability coefficient was to evaluate the uniformity of the stress field. The expression of the stress variability coefficient is as follows:

(1) CV=sd(σ_field)/average(σ_field)

where sd() and average() are the standard deviation and average value of von Mises stress in the central zone, respectively. A successful biaxial tensile specimen requires minimum CV.

(ii) Stress Concentration Factor

The biaxial tensile specimens can be divided into central section and non-central section, where the failure of specimen occurs in the central section. A maximum stress failure criterion is proposed to evaluate biaxial tensile failure. The mathematical expression of maximum failure stress criterion is as follows:

(2) $$\begin{array}{l} k=(\sigma_{\mathrm{ult},\mathrm{cen}}-\sigma_{\max,\mathrm{cen}})/(\sigma_{\mathrm{ult},\mathrm{non}}-\sigma_{\max,\mathrm{non}})\\ \sigma_{\mathrm{ult},\mathrm{non}}=\sigma_{\mathrm{ulten},\mathrm{non}}+\sigma_{\mathrm{ultadd},\mathrm{non}}\;\mathrm{layer}\;\mathrm{of}\;\mathrm{non-central}\;\mathrm{region}=\mathrm{two}\;\mathrm{layers}\\ \sigma_{\mathrm{ult},\mathrm{cen}}=\sigma_{\mathrm{ulten},\mathrm{cen}}\;\mathrm{layer}\;\mathrm{of}\;\mathrm{central}\;\mathrm{region}\;=\;\mathrm{one}\;\mathrm{layer} \end{array}$$

where k is the maximum failure stress criterion factor, σ_max,cen_ represents maximum in-plane principal stress in central section under biaxial tension, and σ_max,non_ represents maximum in-plane principal stress in non-central section under biaxial tension. σ_ult,non_ denotes failure strength in the non-center region, σ_ult,cen_ denotes failure strength in center region. σ_ulten,non_ represents the failure strength of envelope material under uniaxial tension. σ_ultadd,non_ represents the failure strength of strengthening material under uniaxial tension. Maximum failure stress factor k should be less than 1 for successful biaxial tensile specimens. 

### 2.2. Material

**Figure 1:**
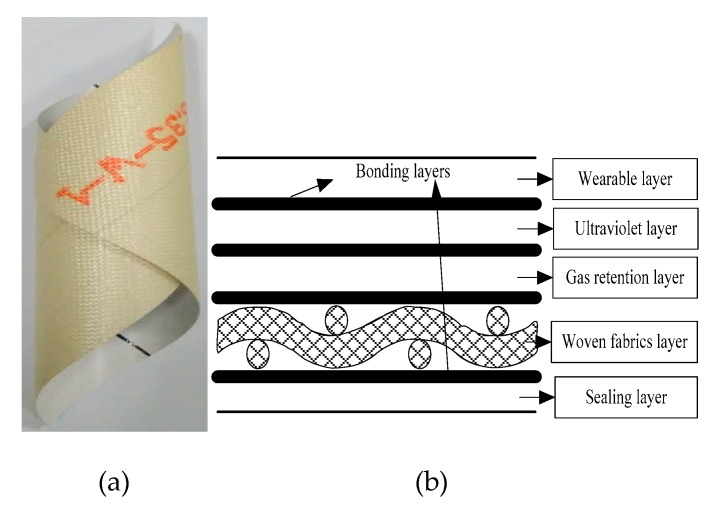
Envelope material [18], (**a**) macro morphology; (**b**) Typical envelope material.

The stratospheric airship envelope Uretek-3216LV studied in this paper is composed of five layers, with a total thickness of 0.21 mm. A surface density of envelope material Uretek-3216LV is 200 g/m^2^. As shown in Figure 1, envelope material contains three parts: external functional layer, the principal force bearing layer, and internal functional layer. External functional layer contains wearable layer, ultraviolet layer, and gas retention layer. The principal force bearing layer is laminated by Vectran fiber.

Uretek-3216LV (material is provided by AVIC Special Vehicle Research Institute, jingmen, China) is a multi-layer flexible laminate material. Tedlar film is selected as the wearable layer, UV layer, and helium barrier. The structural layer is the principal bearing layer. The structural layer is made of high strength Vectran fiber plain woven fabric. Ethylene vinyl alcohol copolymer is selected as the sealing layer. The five layers are thermal sealed together by thermoplastic polyurethane under the temperature of 230 °C and humidity of 50%. 

Table 1 shows the mechanical properties of envelope materials Uretek-3216LV [29]. Because the thickness of the envelope material is less than its plane size, it can be considered as a two-dimensional material.

## 3. Biaxial Specimen Shape and Analysis Conditions

### 3.1. Biaxial Specimen Shape

**Figure 2:**
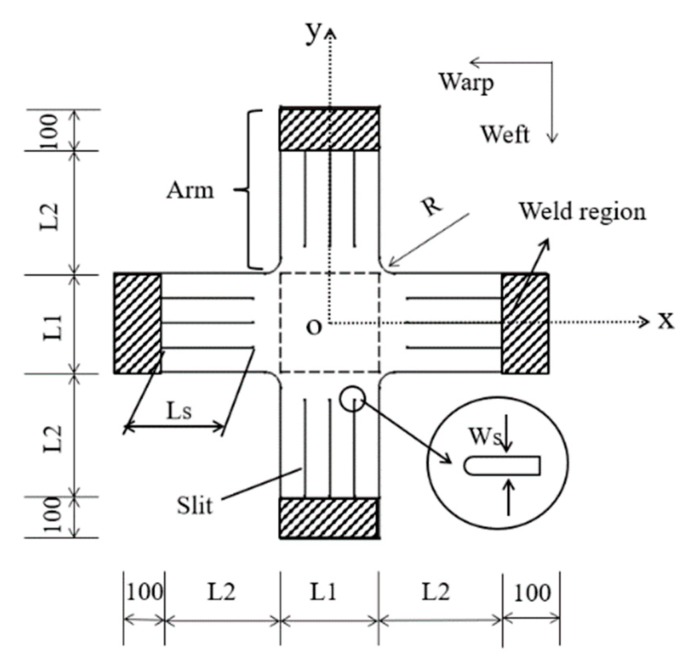
Geometry of base cruciform specimen (Units: mm).

Figure 2 shows the basic design of the biaxial specimen. In this paper, point o is the origin of the coordinate. L1 is the width of the arms, and L2 is the length of the arms. It is assumed that the arm lengths in x and y directions are equal. Ls is the length of the slits and Ws is the width of the slits. R is the corner radius. The length of the clamped regions is 100 mm. The dotted box represents the central test region. The base biaxial specimen is single layer, except the clamped regions is double layer.

**Figure 3:**
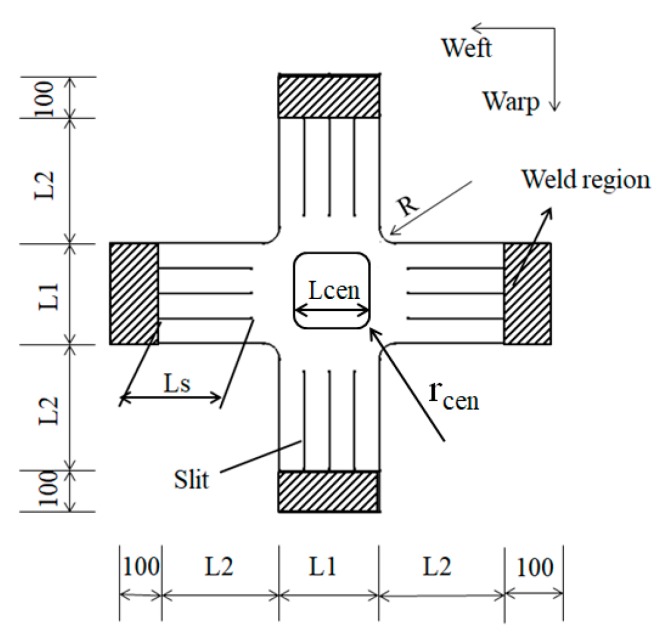
Geometry of modified cruciform specimen (Units: mm).

A modified cruciform specimen comprised of two layers is developed in order to ensure that the initial failure of the biaxial specimen occurs in the central region. Figure 3 shows the geometry of modified cruciform specimen. The modified biaxial specimen has two layers, except the central test regions has one layer. The central black box represents the test section. L_cen_ is the length of the central region and r_cen_ is the corner radius of the central region.

In order to ensure that the initial failure of the biaxial specimen occurs in the central region, it is usually necessary to paste reinforcing materials to the arm. However, the effect of elastic modulus of different materials and dimensions of the central region on stress and stress concentration factor in the central region is less considered. In the following section, the influence of different factors (corner radius, number of slits, slit length, central region length, corner radius of central region) on the stress field in the center of biaxial tension is studied.

### 3.2. Analysis Conditions

Table 2 shows the analysis conditions for finite element model (FEA). The nominal stress ratios chosen were 1:0, 1:1, 2:1, and 4:1. The tensile load of clamped region was 50 N/mm under stress ratio 1:1. The underlined values indicate the standard conditions for FEA. The thickness of the strengthening material is 0.21 mm in the finite element model. The elastic modulus and failure strength are assumed to be n (n = 0.6, 0.8, 1) times of the envelope material. The factors in this study are independent of each other.

Software ABAQUS6.14 was used in finite element analysis. Due to the dimension of envelope material in thickness direction being far smaller than the dimension in plane, it can be regarded as a two-dimensional (2D) plane stress state.

Firstly, the geometric features of the cruciform were precisely reappeared in the software. Table 1 shows the mechanical behavior of envelope materials. 

Secondly, the specimen was meshed on a global seed interval of 2.5 mm. The mesh density of the corner radius is added in order to accurately capture the stress of corner radius. The element type of central region is quad and the element type of non-central region is quad-dominated. Element type S4R was applied in FEA.

Table 3 presents the boundary conditions for numerical model of biaxial tension. The numerical model of biaxial tension in this paper is assumed to be a plane stress model, so the degrees of freedom in out-of-plane direction (z direction) and rotation directions are all zero.

Eventually, the tensile load was applied at the cruciform specimen. The stress ratios of 1:0, 1:1, 2:1, and 4:1 are 50 N/mm:0, 50 N/mm:50 N/mm, 50 N/mm:25 N/mm, and 50 N/mm:12.5 N/mm, respectively.

### 3.3. Mesh Sensitivity Analysis

A limited mesh sensitivity analysis was carried out in order to investigate the effect of mesh density on stress. The standard specimen was chosen for FEA. The tensile load was applied at the cruciform under load stress ratio 1:1 was 50 N/mm:50 N/mm. Three different element sizes were used in the cruciform specimen. The three specimens were meshed on a global seed interval of were 1.25 mm, 1.875 mm, and 2.5 mm, respectively. To enhance the reliability of the model, the number of corner radius was determined to be 21, 32, and 42, respectively. Table 4 shows the number results. As the number of grids increases, the stress change is controlled within 8%, so it can be considered that the mesh number setting was reasonable. The element number in reference 1 was chosen for next FEA.

## 4. Results and Discussion

### 4.1. Effects of the Central Region Length and Central Region Corner Radius

**Figure 4:**
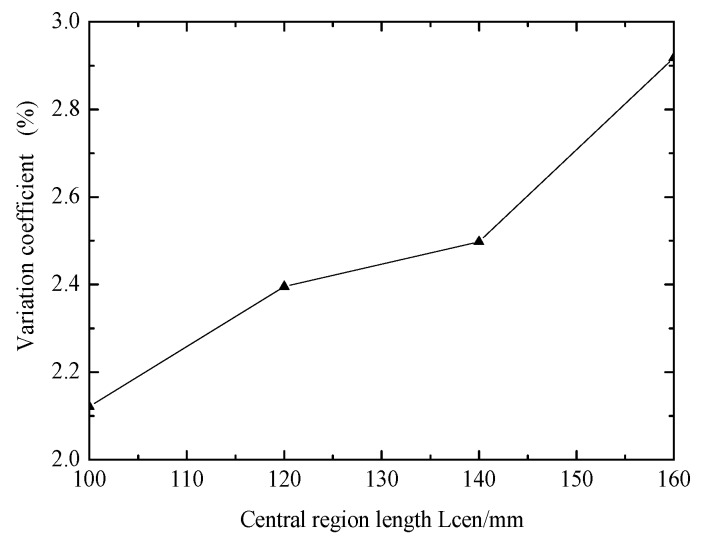
Effect of central region length on stress in central region.

Figure 4 shows the effect of central region length on stress in central region. Variation coefficient increases with increasing central region length. The variation coefficient increased from 2.12% to 2.92%. Biaxial specimens are required to have a large central region and von Mises stress as uniform as possible. Therefore, in the actual design, it is necessary to consider both the test length and the variation coefficient of the central area synthetically.

**Figure 5:**
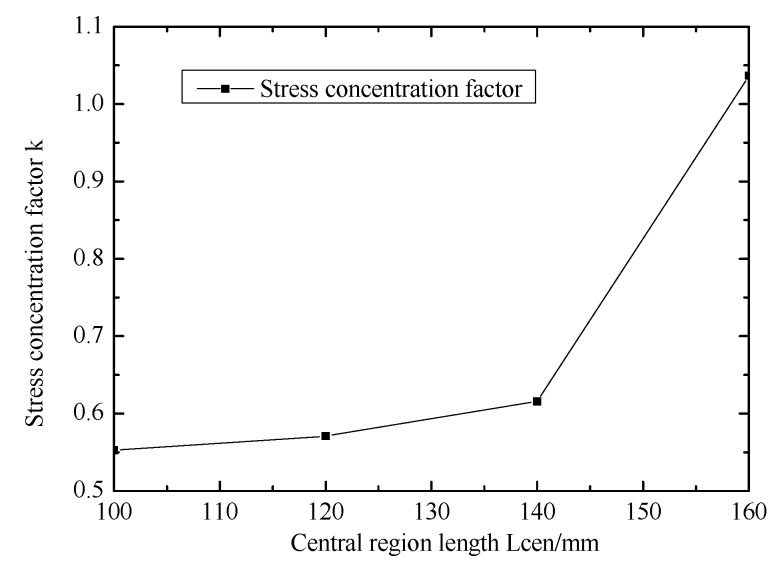
Effect of central region length on stress concentration factor.

Figure 5 shows the effect of central region length on stress concentration factor. Stress concentration factor (k) increases with increasing central region length. the effective center region length of biaxial specimens is no more than 140 mm, k is less than 1, so it is considered to be valid. However, k is more than 1 when the center region length is 160mm, it is considered to be invalid.

**Figure 6:**
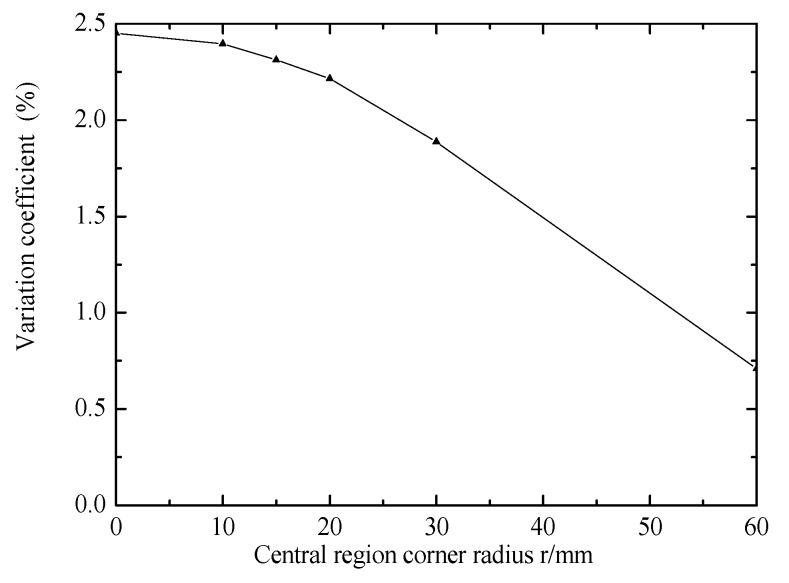
Effect of central region corner radius on stress in central region.

Figure 6 shows the effect of central region corner radius on stress in central region. The coefficient of variation decreases with the increase of the central region corner radius. When the radius of the central region is 60 mm (the central region is circular), the minimum variation coefficient is 0.71%. Increasing the central region corner radius can reduce the variation coefficient.

**Figure 7:**
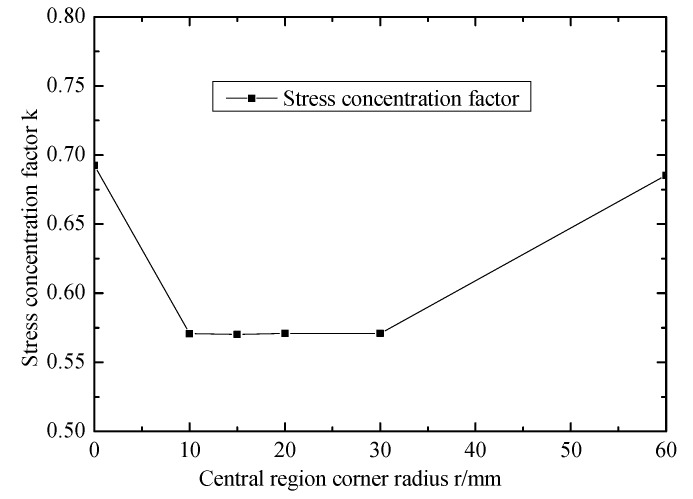
Effect of central region corner radius on stress concentration factor.

Figure 7 shows the effect of central region corner radius on stress concentration factor. The central region corner radius is 0 mm and 60 mm, k is less than 1, so the initial failure occurs in the center region. k in the limit state of r is larger than that in other conditions.

### 4.2. Effect of Modulus of Strengthening Material

**Figure 8:**
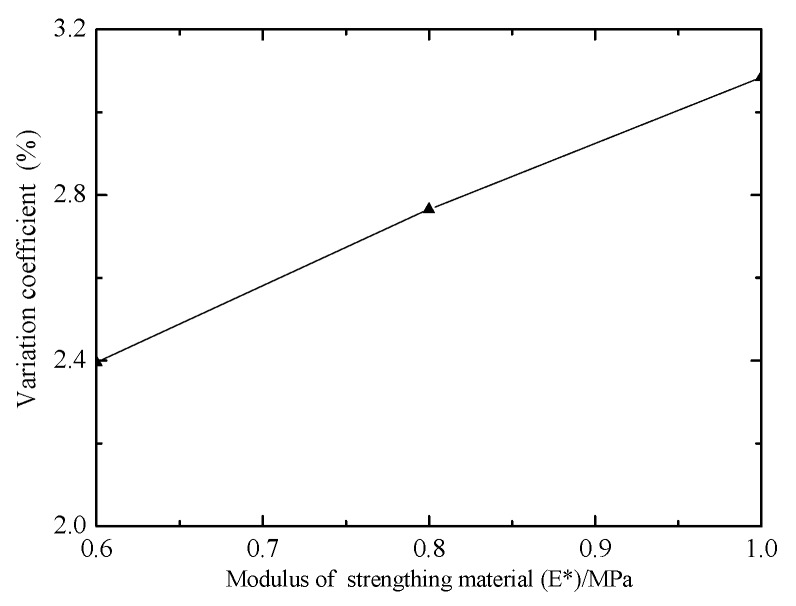
Effect of modulus of strengthening material on stress in central region.

The effect of modulus of strengthening material (E*) on the stress in central region is shown in Figure 8. The variation coefficient increases with the increasing of E*. The biaxial tension specimens require the stress in the central region to be as uniform as possible, so the appropriate modulus of strengthening material is selected to meet the design requirements of the cruciform specimens.

**Figure 9:**
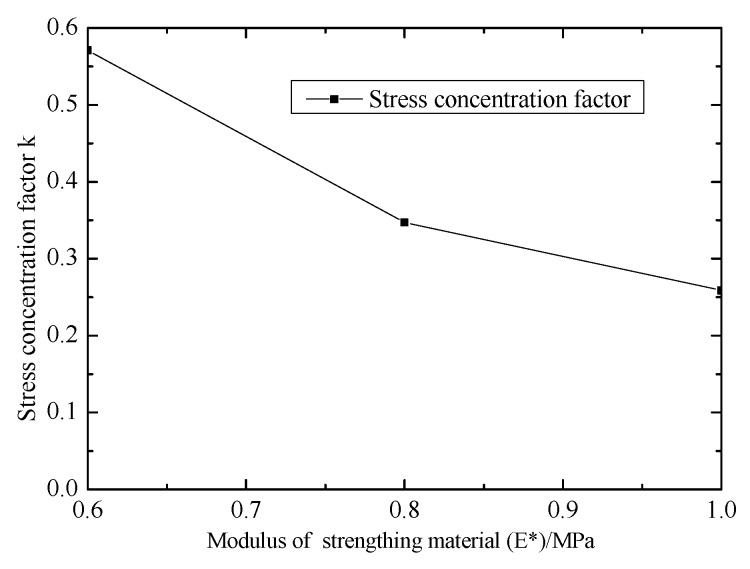
Effect of modulus of strengthening material on stress concentration factor.

Figure 9 shows the effect of modulus of strengthening material (E*) on stress concentration factor. k decreases with increasing E*. k decreased from 0.57 to 0.26. k is lower than 1, so the initial failure will occur in the central region.

### 4.3. Effect of Stress Ratios

**Figure 10:**
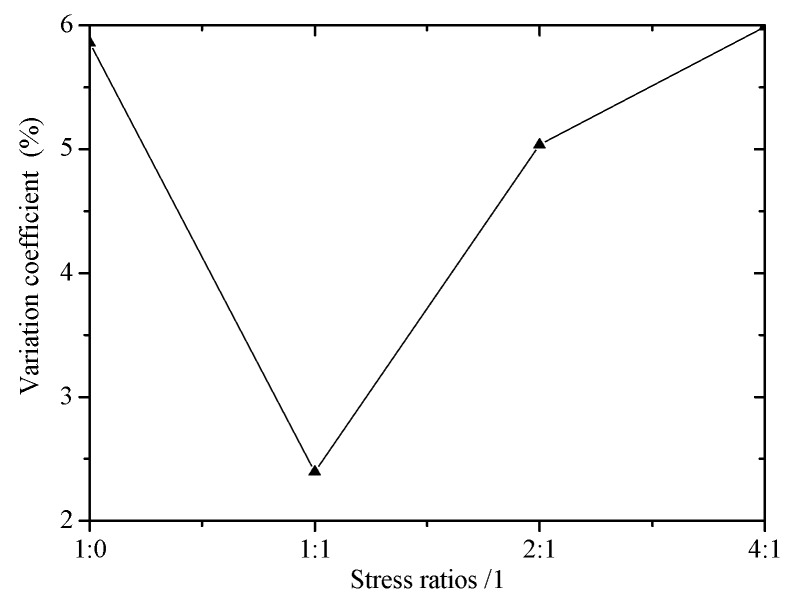
Effect of stress ratios on stress in central region.

Figure 10 shows the effect of stress ratios on stress in central region. The stress ratios (warp:weft) of 1:0, 1:1, 2:1, 4:1 are 50 N/mm:0 N/mm, 50 N/mm:50 N/mm, 50 N/mm:25 N/mm, 50 N/mm:12.5 N/mm respectively. The variation coefficient at the stress ratio of 1:1 is smaller than that of other stress ratios. The minimum variation coefficient is 2.40% and the maximum variation coefficient is 5.99%.

**Figure 11:**
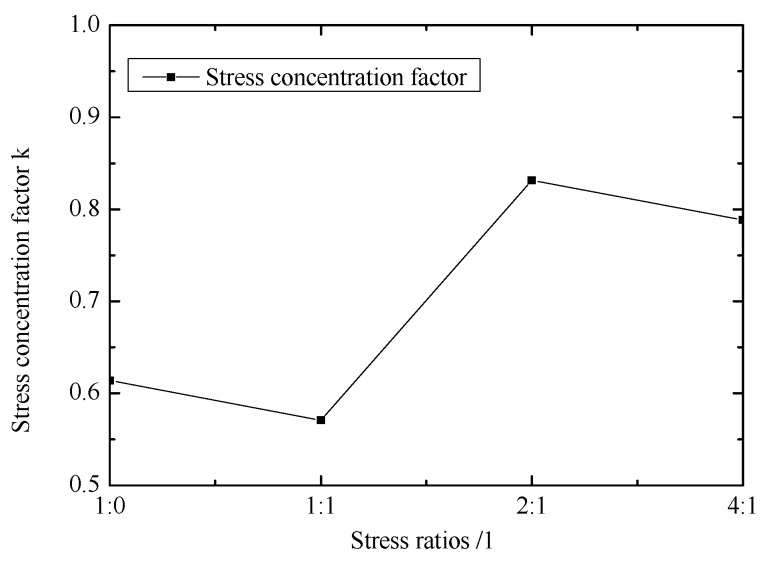
Effect of stress ratios on stress concentration factor.

Figure 11 shows the effect of stress ratios on stress concentration factor. k under the stress ratio of 1:1 is smaller than k in other stress ratios. k is lower than 1, so the initial failure will occur in central region.

### 4.4. Optimum Specimen

Different evaluation criteria can obtain different optimized samples. The order of importance of the three parameters in this paper is as follows: stress concentration factor > coefficient of variation. Table 5 shows the optimum specimen size. Parameters L1, L2, R, N, Ws, and Ls are the same as in Table 2. The tensile load was applied at the cruciform under load stress ratio 1:1 was 50 N/mm: 50 N/mm. the k of two type specimens are both less than 1, so the initial failure will occur in central region. The CV value of the optimized specimen is smaller than that of the original specimen, which means that the stress in the central region of the optimized specimen is more uniform than that in the original specimen.

**Figure 12:**
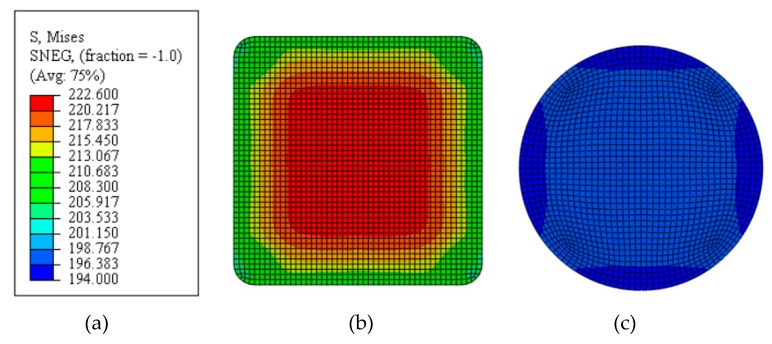
Stress contour diagram of central region with two type specimens. (**a**) stress contour bar; (**b**) original specimen; and, (**c**) optimum specimen.

Figure 12 provides the stress contour diagram with two type specimens. The stress in the center region of the optimized specimen is less than that in the original specimen. The stress uniformity region of the optimized specimen is better than that of the original specimen.

## 5. Conclusions

In this paper, a theoretical model is developed to study the strengthening material and geometric parameters on the mechanical behavior of the biaxial tensile specimens. The method can be used to guide the design of cruciform specimens and obtained reliable biaxial strength in operated condition, hence providing more accurate design for envelope structure. The obtained results can be summarized, as follows:

(1) The central region length L_cen_ has great influence on the variation coefficient and stress concentration factor. The variation coefficient and stress concentration factor increase with increasing of L_cen_.
(2) The influence of central region corner radius r on variation coefficient and stress concentration factor are different in the central region. With the increasing of r, the coefficient of variation decreases and the stress concentration factors are lower than 1.
(3) Modulus of strengthening material E* has great influence on the coefficient of variation and stress concentration factor. Average stress increases slightly with the increase of E* and the variation coefficient increases with increasing of E*, while stress concentration factor decreases with increasing of E*.
(4) Stress ratios (S_x_:S_y_) has great influence on the variation coefficient and stress concentration factor. The variation coefficient and stress concentration factor are the smallest when the stress ratio is 1:1.
(5) In this paper, under the given design criteria, the optimal shape and the original shape can be realized in the central region. The coefficients of variation of the optimized shape are less than those of the original shape.

## Figures and Tables

**Table 1:** Mechanical parameters of envelope Uretek-3216LV [29].

Direction	Warp Modulus (MPa)	Weft Modulus (MPa)	Poisson’s Ratio	Shear Modulus (MPa)	Warp Failure Strength (MPa)	Weft Failure Strength (MPa)	Shear Failure Strength (MPa)
value	4092	3180	0.35	138	322	300	193

**Table 2:** Analysis conditions for finite element model (FEA) (underlined: standard conditions).

Variable factors	Value	Note
Stress ratios S_x_: S_y_ (warp: weft)	1:0, 1:1, 2:1, 4:1	-
Arm width L1 (mm)	160	-
Arm length L2 (mm)	160	
Corner radius R (mm)	15	
Number of slits	3	
Slit width Ws (mm)	1	
Slit length Ls (mm)	155	
Central region length Lcen (mm)	100, 120, 140, 160	Clamped region: two layers (envelope material); Central region: single layer Other regions: two layers (envelope material + strengthening material)
Central region corner radius r (mm)	0, 10, 20, 30, 60	
Modulus of arm strengthening material E* (MPa)	0.6E, 0.8E, 1E (E: Modulus of envelope Material)	

**Table 3:** Boundary conditions for the in-plane stress finite element model.

Reference	Node Coordinated	Displacement In	
		x	y
1	x = y = 0	0	0
2	x = 0	0	Free
3	y = 0	Free	0

**Table 4:** Mesh sensitivity analysis on stress.

Reference	Number of Elements	Maximum Stress of Global Specimen (MPa)	Maximum Stress in Central Region (MPa)
1	34,899	345	223
2	62,143	354	223
3	140,278	370	223

**Table 5:** Parameter value for the optimum specimen (load ratio = 1:1).

Type	L_cen_ (mm)	R (mm)	E* (MPa)	k	CV
Original	120	10	0.6E	0.57	2.40%
Optimum	100	50	0.6E	0.94	0.51%

## References

[1] Ilcev S.D. (2011). Stratospheric communication platforms as an alternative for space program. Aircr. Eng. Aerosp. Technol..

[2] Smith M., Rainwater L. Applications of scientific ballooning technology to high altitude airships. Proceedings of the AIAA’s 3rd Annual Aviation Technology, Integration, and Operations (ATIO) Forum.

[3] Meng J., Li P., Ma G., Du H., Lv M. (2016). Tearing Behaviors of Flexible Fiber-Reinforced Composites for the Stratospheric Airship Envelope. Appl. Compos. Mater..

[4] Androulakakis S.P., Judy R. Status and Plans of High Altitude Airship (HAATM) program. Proceedings of the Aiaa Lighter-Than-Air Systems Technology Conferences.

[5] Zhai H., Euler A. Material Challenges for Lighter-Than-Air Systems in High Altitude Applications. Proceedings of the Aiaa Atio & Lighter-Than-Air Sys Tech & Balloon Systems Conferences.

[6] Komatsu K., Sano M.A., Kakuta Y. (2013). Development of High Specific Strength Envelope Materials. Jpn. Soc. Aeronaut Space Sci..

[7] Stockbridge C., Ceruti A., Marzocca P. (2012). Airship Research and Development in the Areas of Design, Structures, Dynamics and Energy Systems. Int. J. Aeronaut. Space Sci..

[8] Meng J., Qu Z., Zhu W., Lv M. (2016). Fatigue Damage Mechanical Model of the Envelope Material for Stratospheric Airships. Appl. Compos. Mater..

[9] Hu J., Gao C., He S., Chen W., Li Y., Zhao B., Shi T., Yang D. (2017). Effects of on-axis and off-axis tension on uniaxial mechanical properties of plain woven fabrics for inflated structures. Compos. Struct..

[10] Meng J., Lv M., Qu Z., Li P. (2017). Mechanical Properties and Strength Criteria of Fabric Membrane for the Stratospheric Airship Envelope. Appl. Compos. Mater..

[11] Roh J.-H., Lee H.-G., Lee I. (2008). Thermoelastic Behaviors of Fabric Membrane Structures. Adv. Compos. Mater..

[12] Chen J., Chen W., Zhang D. (2014). Experimental study on uniaxial and biaxial tensile properties of coated fabric for airship envelopes. J. Reinf. Plast. Compos..

[13] Chen J., Chen W., Wang M., Ding Y., Zhou H., Zhao B., Fan J. (2017). Mechanical Behaviors and Elastic Parameters of Laminated Fabric URETEK3216LV Subjected to Uniaxial and Biaxial Loading. Appl. Compos. Mater..

[14] Longbin L., Mingyun L., Houdi X. (2014). Tear strength characteristics of laminated envelope composites based on single edge notched film experiment. Eng. Fract. Mech..

[15] De Camargo F.V., Pavlovic A. (2017). Fracture Evaluation of the Falling Weight Impact Behaviour of a Basalt/Vinylester Composite Plate through a Multiphase Finite Element Model. Key Eng. Mater..

[16] Fragassa C., Pavlovic A., Santulli C. (2018). Mechanical and impact characterisation of flax and basalt fibre vinylester composites and their hybrids. Compos. Part B Eng..

[17] Chen S., Ding X., Fangueiro R., Yi H., Ni J. (2010). Tensile behavior of PVC-coated woven membrane materials under uni- and bi-axial loads. J. Appl. Polym. Sci..

[18] Qu Z., He W., Lv M., Xiao H. (2018). Large-Strain Hyperelastic Constitutive Model of Envelope Material under Biaxial Tension with Different Stress Ratios. Materials.

[19] Hanabusa Y., Takizawa H., Kuwabara T. (2013). Numerical verification of a biaxial tensile test method using a cruciform specimen. J. Mater. Process. Technol..

[20] Shi T., Chen W., Gao C., Hu J., Zhao B., Wang P., Wang M. (2018). Biaxial strength determination of woven fabric composite for airship structural envelope based on novel specimens. Compos. Struct..

[21] Zhang Y., Song X., Zhang Q., Lv H. (2014). Fracture failure analysis and strength criterion for PTFE-coated woven fabrics. J. Compos. Mater..

[22] Hannon A., Tiernan P. (2008). A review of planar biaxial tensile test systems for sheet metal. J. Mater. Process. Technol..

[23] Xiao R. (2019). A Review of Cruciform Biaxial Tensile Testing of Sheet Metals. Exp. Tech..

[24] Kwon H.J., Jar P.-Y.B., Xia Z. (2005). Characterization of Bi-axial fatigue resistance of polymer plates. J. Mater. Sci..

[25] Geiger M., Hußnätter W., Merklein M. (2005). Specimen for a novel concept of the biaxial tension test. J. Mater. Process. Technol..

[26] Demmerle S., Boehler J. (1993). Optimal design of biaxial tensile cruciform specimens. J. Mech. Phys. Solids.

[27] Yu Y., Wan M., Wu X.-D., Zhou X.-B. (2002). Design of a cruciform biaxial tensile specimen for limit strain analysis by FEM. J. Mater. Process. Technol..

[28] Makris A., Vandenbergh T., Ramault C., Van Hemelrijck D., Lamkanfi E., Van Paepegem W. (2010). Shape optimisation of a biaxially loaded cruciform specimen. Polym. Test..

[29] Chen J., Chen W., Zhao B., Yao B. (2015). Mechanical responses and damage morphology of laminated fabrics with a central slit under uniaxial tension: A comparison between analytical and experimental results. Constr. Build. Mater..

